# *Streptococcus agalactiae* infective endocarditis in Canada: a multicenter retrospective nested case control analysis

**DOI:** 10.1186/s12879-021-06997-6

**Published:** 2022-01-04

**Authors:** Torrance Oravec, S. Annie Oravec, Jennifer Leigh, Liam Matthews, Bahareh Ghadaki, Dominik Mertz, Peter Daley, Anjali Shroff

**Affiliations:** 1 Division of Infectious Diseases, Department of Medicine, University of British Columbia, 328C Heather Pavilion E, 2733 Heather St. Vancouver, Vancouver, BC V5Z 3J5 Canada; 2 Division of Respirology, Department of Medicine, University of British Columbia, Vancouver, BC Canada; 3 Division of Internal Medicine, Department of Medicine, University of Ottawa, Ottawa, ON Canada; 4 Division of Internal Medicine, Department of Medicine, Memorial University of Newfoundland, St. John’s, NL Canada; 5 Division of Infectious Diseases, Department of Medicine, Halton Healthcare, Oakville, ON Canada; 6 Division of Infectious Diseases, Department of Medicine, McMaster University, Hamilton, ON Canada; 7 Division of Infectious Diseases, Department of Medicine, Memorial University of Newfoundland, St. John’s, NL Canada

**Keywords:** *Streptococcus agalactiae*, Group B *Streptococcus*, Infective endocarditis, Case control, Retrospective

## Abstract

**Background:**

Infective endocarditis (IE) caused by *Streptococcus agalactiae* (GBS) is increasingly reported and associated with an aggressive course and high mortality rate. Existing literature on GBS IE is limited to case series; we compared the characteristics of patients with GBS IE to patients with GBS bacteremia without IE to identify risk factors for development of IE.

**Methods:**

A nested case–control study in a cohort of adult patients with GBS bacteremia over a 18-year period was conducted across seven centres in three Canadian cities. A chart review identified patients with possible or definite IE (per Modified Duke Criteria) and patients with IE were matched to those without endocarditis in a 1:3 fashion. Multivariate analyses were completed using logistic regression.

**Results:**

Of 520 patients with GBS bacteremia, 28 cases of possible or definite IE were identified (5.4%). 68% (19/28) met criteria for definite IE, surgery was performed in 29% (8/28), and the overall in-hospital mortality rate was 29% (8/28). Multivariate analysis demonstrated that IE was associated with injection drug use (OR = 19.6, 95% CI = 3.39–111.11, p = 0.001), prosthetic valve (OR = 11.5, 95% CI = 1.73–76.92, p = 0.011) and lack of identified source of bacteremia (OR = 3.81, 95% CI = 1.24–11.65, p = 0.019).

**Conclusions:**

GBS bacteremia, especially amongst people who inject drugs, those with prosthetic valves, and those with no apparent source of infection, should increase clinical suspicion for IE.

## Background

Serious systemic infections due to *Streptococcus agalactiae* (Group B *Streptococcus*—GBS) in non-pregnant adults are increasingly reported [1–4]; a recent multinational population-based assessment demonstrated increased incidence for invasive GBS infection, driven by an increase in adults over 60 [5]. GBS is known to cause invasive disease in pregnancy, the peripartum period, and in neonates [6], but other disease manifestations include pneumonia, skin and soft tissue infection, osteomyelitis, joint infection, abscess, meningitis, endocarditis and bacteremia without focus [2, 3]. Numerous risk factors for the development of invasive GBS disease in non-pregnant adults have been identified, particularly diabetes mellitus, but also other immunocompromising states, active malignancy, and advanced age [2, 3, 7–9].

GBS has been increasingly been reported as a cause of Infective endocarditis (IE) and is of particular interest because of its association with an aggressive course, highly destructive effect on valvular tissue, and high mortality rate [5, 10–13]. Description of GBS IE is limited to case series which have examined the epidemiology, natural history, and complications of this disease, and provide insight into how the disease has been treated in specific circumstances [4, 10, 13, 14]. More recent analyses have demonstrated that GBS is associated with an aggressive IE phenotype in comparison to other *Streptococcus* species, and is likely the most common beta-hemolytic Streptococcus causing IE [12]. No studies have examined risk factors for the development of IE amongst individuals with invasive GBS disease.

We present a retrospective, nested case–control analysis of GBS IE and GBS bacteremia, to compare these groups and to describe risk factors for the development of IE.

## Methods

### Patient population

Our study was conducted across seven hospitals in three Eastern Canadian cities (Hamilton and Niagara, both in Ontario, and St John’s in Newfoundland). Patient records from January 1, 2000 to December 31, 2018 were reviewed. Ethics approval was obtained from local Research Ethics Boards with waiver of patient consent.

All patients of at least 18 years of age that had blood cultures positive for *Streptococcus agalactiae* were identified through local microbiology labs, and medical records were screened for the diagnosis of IE. Cases were identified if they met the Modified Duke Criteria (chosen given widespread use in previous literature) for possible or definite IE, and controls either did not meet criteria or had the diagnosis rejected [15]; some cases were rejected despite meeting Modified Duke Criteria due to presence of a firm alternate diagnosis or resolution of symptoms within four days. For each case we identified three controls, matched by study site and proximity of bacteremia in time to the corresponding case. It was felt that matching cases to controls who presented to the same hospital at a similar time would help to control for resource availability and management strategies.

### Data collection and outcomes

Data was collected using a standardized case report form and de-identified prior to analysis. Risk factors for GBS IE identified in previous case series were included, as were generally accepted risk factors for IE and for invasive GBS infections [10, 12, 15]. Outcomes were compared including mortality, congestive heart failure, cardiac conduction abnormalities, neurological complications, and need for valvular surgery.

### Statistical analysis

We compared cases and controls using a paired t-test for continuous variables and Pearson’s test for categorical variables. We used Cox-Snell binary logistic regression (conditional, forward step-wise) for multivariate analysis, including all variables with p < 0.05 from univariate analysis. Statistical analysis was performed on SPSS version 25 (IBM, USA). Cases with missing data were not included in risk factor analysis, nor were matched controls.

## Results

Five hundred twenty patients with a total of 827 positive blood cultures were included. 28/520 patients (5.4%) met the case definition of IE (19 definite IE, 9 possible IE). A single case was identified during the years 2000–2010, with the remaining 27 cases identified from 2011 to 2018. 84 matched controls were identified.

Demographic and baseline clinical characteristics of cases and controls are presented in Table 1. Cases and controls were similar in terms of age, sex and comorbidities. Cases were significantly more likely to have valvular disease at baseline than controls (35.7 vs 8.3%; p < 0.001), more likely to have a prosthetic valve (17.9 vs 2.4%; p = 0.003), more likely to have a history of injection drug use (25.0 vs. 2.4%; p < 0.001), and more likely to have a history of alcohol use disorder (14.3 vs 1.2%; p = 0.004).

**Table 1:** Demographic and baseline clinical characteristics of Group B *Streptococcus* infective endocarditis cases and matched controls

	Cases (N = 28)	Controls (N = 84)	(Pearson 2-sided Chi-square, or 2-tailed t test)
Age in years (mean ± SD)	59.0 ± 16.8	65.4 ± 15.5	P = 0.066
Females	14 (50.0%)	37 (44.0%)	P = 0.58
Non-valvular cardiac disease	11 (39.3%)	34 (40.5%)	P = 0.84
Atrial fibrillation	4 (14.3%)	17 (20.2%)	P = 0.49
Coronary artery disease	9 (32.1%)	21 (25.0%)	P = 0.46
Congestive heart failure	2 (7.1%)	8 (9.5%)	P = 0.70
Valvular disease	10 (35.7%)	7 (8.3%)	P < 0.001
Cardiac device	0	4 (4.8%)	P = 0.24
Prosthetic valve	5 (17.9%)	2 (2.4%)	P = 0.003
Diabetes	12 (42.9%)	43 (51.2%)	P = 0.48
Active malignancy	3 (10.7%)	8 (9.5%)	P = 0.86
Immunocompromised*	3 (10.7%)	12 (14.3%)	P = 0.68
Pregnant	1 (3.6%)	1 (1.2%)	P = 0.41
Post-partum	0	3 (3.6%)	P = 0.31
Cirrhosis	3 (10.7%)	6 (7.1%)	P = 0.55
Genitourinary disease	4 (14.3%)	16 (19.0%)	P = 0.57
Chronic kidney disease	6 (21.4%)	16 (19.0%)	P = 0.78
Hemodialysis	1 (3.6%)	5 (6.0%)	P = 0.63
Injection drug use	7 (25.0%)	2 (2.4%)	P < 0.001
Alcohol use disorder	4 (14.3%)	1 (1.2%)	P = 0.004

*HIV/AIDS, neutropenia, organ transplantation, immunoglobulin deficiency, immunosuppressive therapy (chemotherapy, radiotherapy, anti-TNF-alpha, DMARDs, or immunosuppressive biologics), splenectomy/asplenia

The clinical course and outcome of cases and controls are detailed in Table 2. Invasive GBS tended to present acutely in both cases and controls, with average days of symptoms before presentation of 4.9 and 4.2, respectively (p = 0.64). Cases did not have significantly shorter time to culture positivity (11.8 vs 15.5 h; p = 0.17), but had a greater number of positive blood culture sets (2.9 vs. 1.7; p < 0.001) as they had more blood culture sets drawn (2.5 vs 2.1; p = 0.048). All cases were community acquired, whereas five controls had nosocomial infections (p = 0.19). Cases were not significantly more likely to have had a recent procedure (10.7 vs 3.6%; p = 0.15). No significant differences were observed in rates of concurrent skin, soft tissue, bone or joint infection, nor rates of indwelling lines. Amongst controls, skin and soft tissue infection was the source in 33 patients, respiratory infection in 9, genitourinary in 13, osteoarticular in 8, and central line associated in 1; 20 controls did not have a source identified.

**Table 2:** Clinical course and outcomes for GBS IE cases and controls

	Cases (N = 28)	Controls (N = 84)	(Pearson 2-sided Chi-square, or 2-tailed t test)
Mean days of symptoms Before diagnosis	4.9 ± 5.9	4.2 ± 7.2	P = 0.64
Mean time to culture Positivity (hours)	11.8 ± 6.2	15.5 ± 13.2	P = 0.17
Mean number of positive Blood culture sets	2.9 ± 2.6	1.7 ± 0.7	P < 0.001
Mean number of blood Culture sets collected	2.5 ± 0.5	2.1 ± 0.4	P = 0.048
Community acquired	28 (100%)	79 (94.0%)	P = 0.19
Concurrent skin or soft tissue infection	8 (28.6%)	34 (40.5%)	P = 0.26
Concurrent bone or joint infection	6 (21.4%)	15 (17.9%)	P = 0.68
Recurrent bacteremia	4 (14.3%)	0	P < 0.001
Source identified	14 (50.0%)	65 (77.0%)	P = 0.006
Recent procedure*	3 (10.7%)	3 (3.6%)	P = 0.15
Indwelling line	1 (3.6%)	3 (3.6%)	P = 0.96
Echo done	24 (85.7%)	38 (45.2%)	P < 0.001
ID consult	21 (75.0%)	27 (32.1%)	P < 0.001
Outcomes			
In hospital mortality	8 (28.6%)	3 (3.6%)	P < 0.001
Mean duration of antibiotics (days)	45.1 ± 40.4	19.9 ± 15.7	P < 0.001

*Any surgeries, invasive procedures in past 3 months, including PICC/central line insertion, endoscopy, suprapubic catheter insertion, C-section, therapeutic abortion

Cases were more likely to have an echocardiogram done than controls (85.7% vs 45.2%; P < 0.001). Ten cases also received transesophageal echocardiography, whereas only four controls did. Four cases did not have an echocardiogram but met Modified Duke’s Criteria for “possible endocarditis); two of these cases died early in the treatment course, and the other two received relatively short courses of therapy. Cases were also more likely to receive consultation from an Infectious Disease specialist (75% vs 32.1%; P < 0.001). Cases were more likely to have recurrent bacteremia, defined as blood cultures positive for GBS following documented negative cultures (14.3% vs 0%; p < 0.001), and were less likely to have a source identified (50% vs 77%; p = 0.006). Patients with GBS IE received 45.1 days of antibiotics on average, while patients without endocarditis received an average of 19.9 days of antibiotics (p < 0.001).

Description of cases is presented in Table 3. Of cases that had echocardiograms performed, 50% had aortic valve infection, with tricuspid valve infection being second most common (33.3%) followed by mitral valve (20.8%). No patients had documented pulmonic valve involvement, and a single patient had both mitral and tricuspid endocarditis. High rates of complications were observed among cases: 32.1% had acute heart failure, 32.1% had neurologic complications (embolic stroke or epidural abscess), 16.7% had valve perforation, 12.5% had an intracardiac abscess, and 10.7% had cardiac conduction system disease. In-hospital mortality was significantly higher in endocarditis cases than in controls (28.6% vs 3.6%; p < 0.001). Surgery was performed in 28.6% of cases, and all patients undergoing surgery survived to discharge.

**Table 3:** Clinical characteristics and complications occurring in GBS IE cases

	Cases (N = 28)
Aortic valve	12 (50%)*
Mitral valve	5 (20.8%)*
Tricuspid valve	8 (33.3%)*
Valve perforation	4 (16.7%)*
Intracardiac abscess	3 (12.5%)*
Congestive heart failure	9 (32.1%)
Conduction disease	3 (10.7%)
Neurological complication	9 (32.1%)
Surgery	8 (28.6%)

*Percentages for valves involved and valvular complications use a denominator of 24, as no echo was done for 4 patients (IE diagnosed without echocardiographic criteria). A single case had both mitral and tricuspid valve involvement

### Multivariate analysis

Results of multivariate analysis are presented in Table 4. One case and three controls were excluded from multivariate analysis because of missing data. Regression proceeded over four cycles, at which point injection drug use (OR for IE = 19.6, 95% CI = 3.39–111.11, p = 0.001), prosthetic valve (OR for IE = 11.5, 95% CI = 1.73–76.92, p = 0.011) and lack of identified source of bacteremia (OR for IE = 3.81, 95% CI = 1.24–11.65, p = 0.019) emerged as significant predictors for the development of IE.

**Table 4:** Multivariate analysis of risk factors for the development of IE amongst patients with GBS bacteremia

Risk factor	OR	95% CI	p value
Injection drug use	19.6	3.39–111.11	0.001
Prosthetic valve	11.5	1.73–76.92	0.011
Recurrent bacteremia	0	N/A	0.99
Source identified	0.26	0.085–0.80	0.019

1 case and 3 controls excluded for missing data

Binary logistic regression (conditional, forward step-wise) included: native valve disease (categorical), injection drug use (categorical), alcohol use disorder (categorical), recent procedure (categorical), prosthetic valve (categorical), time to positivity (continuous), community acquired (categorical), recurrent bacteremia (categorical), source identified (categorical).

Four iterations.

Step 4 R^2^ = 0.305 (Cox and Snell).

Excluded from model (p > 0.05): native valve disease (categorical), alcohol use disorder (categorical), recent procedure (categorical), time to positivity (continuous), community acquired (categorical).

Model predicts 95.1% of controls and 55.6% of cases.

## Discussion

Our retrospective nested case control study found that injection drug use, presence of a prosthetic valve, and lack of apparent source were all risk factors for IE among patients with GBS bacteremia, and provides a novel perspective on the clinical characteristics of GBS IE. Mortality amongst cases was significantly higher than in controls, and rates of systemic complications were similarly high. Interestingly, many previously identified risk factors for the development of invasive GBS disease (diabetes, immunocompromise, malignancy, advanced age) were not significant predictors of IE, illustrating the distinct pathophysiology of endovascular infections [3, 8, 9].

Amongst our study population, 5.4% of patients with *Streptococcus agalactiae* blood stream infections developed IE, which is roughly consistent with previously reported incidence of IE amongst GBS bacteremia (8.5%) [13] and amongst invasive GBS infections overall (3.0–10.5%) [1, 3]. During the eighteen years of our study, the annual incidence of GBS IE increased, with 27/28 cases (96.4%) occurring in the later seven years, a trend which has been observed at other centres [2, 3, 13, 14]. The overall incidence of invasive GBS disease has also increased across populations in Australia, Canada, Denmark, Sweden, Finland and the UK [5, 6, 16]. Population level surveillance explains this increase based on aging [5], but higher rates of comorbidities (diabetes, immunosuppression and malignancy) [8, 9], reduced physical capacity, and altered host immune response may also contribute [7].

Our GBS IE cases demonstrated a left side predominance, which has been reported consistently across multiple studies [4, 10, 12, 13]. We observed high rates of complications, similar to previous reports [12]. The tendency for GBS to cause systemic embolism has been attributed to the tendency towards large, friable vegetations, which have themselves been related to the capacity for GBS to bind fibrinogen and platelets [17]. A similar mechanism of virulence has been proposed for the pathogenesis of the less common entity of *Streptococcus pyogenes* IE [18].

We observed an in-hospital mortality rate among cases of 28.6%. Mortality was 85% in the pre-antibiotic era [4], and mortality reported in case series from the 1960’s to the 1990’s ranged from 40.7 to 85.7%, though many of these series involved less than 10 patients [4, 10, 11, 13, 14, 19]. Sambola et al*.* conducted a review of 115 cases of GBS IE and described mortality as 45% between 1962 and 1979, and 34% between 1980 and 1998. In studies that report data collected after the year 2000, mortality has ranged from 20 to 33.3%, with the relative reduction in mortality attributed to increased recognition, improved diagnosis, and early surgical intervention [12, 20]. The rate of surgical intervention previously reported for GBS IE is highly variable (14.3–83.3%), and the relatively low mortality rate in our study is not clearly related to differences in rates of surgical intervention [4, 20]. The lower mortality rate in our study may be related to an increased proportion of young patients with intravenous drug use as a risk factor (as opposed to older, comorbid diabetic patients described in other studies) [10].

The most novel aspect of our study is that the nested case–control design allows assessment of factors which may predispose individuals who have GBS bacteremia towards developing IE, a type of analysis that has not previously been reported. Based on our multivariate analysis, independent predictors for the development of IE include injection drug use, having a prosthetic valve, and not having a clearly identified source of bacteremia. This is not particularly surprising, as injection drug use and prosthetic valves are so well recognized as conditions predisposing to IE that they are included in the Modified Duke Criteria [15]. Of note, a greater proportion of people who inject drugs were present in our study than have been reported in most other GBS IE case series (two of the seven patients described by Gallagher et al*.*, otherwise rates have been 3.2–8.3%) [4, 12–14]. While local variations in demographic factors may contribute, our data are the most contemporary available, and our relatively high proportion of people who inject drugs is likely due, at least in part, to the ongoing opiate epidemic in Canada [21].

Conditions which have been previously associated with invasive GBS (diabetes, increased age, active malignancy and immunosuppression) did not contribute to risk of developing IE in our study [5, 7, 8]. This may be due to the fact that these conditions predispose to GBS bacteremia but do not impact risk of IE.

Limitations of our design include retrospective data collection, which may have misidentified cases and controls based on missing data in the medical records. While our study does have a relatively high number of cases compared to previous reports, our case numbers were still limited; larger numbers would allow for more robust conclusions to be drawn. Our limited case numbers also prevented determination of differences in outcome due to antimicrobial choice. Our study was conducted exclusively in Canada, and generalizability to other regions is not assured. Not all cases and controls received an echocardiogram, so additional cases may have been identified if every patient underwent the same investigations, though we did not observe any recurrence of bacteremia in controls who had previously been admitted for GBS bacteremia without IE. Additionally, our use of Modified Duke Criteria to define cases may not reflect pragmatic treatment decisions; two of our cases did not receive echocardiograms, and were treated with short courses of therapy, indicating clinicians are not strictly following these criteria when making treatment decisions. Similarly, ID consultation was more likely in cases, and may have led to a more thorough diagnostic evaluation leading to a diagnosis of IE.

## Conclusions

*Streptococcus agalactiae* is an increasingly common cause of infective endocarditis and, coupled with a high mortality rate and markedly destructive phenotype, warrants consideration as a cause of IE. Presence of GBS bacteremia, especially amongst people who inject drugs, those with pre-existing valvular disease, and those with no apparent source of infection, should prompt clinicians to have a high suspicion for IE.

## Data Availability

The datasets used and/or analysed during the current study are available from the corresponding author on reasonable request.

## Abbreviations

DMARD: Disease modifying anti-rheumatic drugs
GBS: Group B *Streptococcus*, *Streptococcus agalactiae*
HIV/AIDS: Human Immunodeficiency Virus/Acquired Immunodeficiency Syndrome
ID: Infectious diseases
IE: Infective endocarditis
